# Machine learning for data integration in human gut microbiome

**DOI:** 10.1186/s12934-022-01973-4

**Published:** 2022-11-23

**Authors:** Peishun Li, Hao Luo, Boyang Ji, Jens Nielsen

**Affiliations:** 1 Department of Biology and Biological Engineering, Chalmers University of Technology, Gothenburg, Sweden; 2 BioInnovation Institute, Ole Maaløes Vej 3, DK2200 Copenhagen, Denmark

**Keywords:** Gut microbiome, Data integration, Machine learning, Precision medicine, Multi-omics

## Abstract

Recent studies have demonstrated that gut microbiota plays critical roles in various human diseases. High-throughput technology has been widely applied to characterize the microbial ecosystems, which led to an explosion of different types of molecular profiling data, such as metagenomics, metatranscriptomics and metabolomics. For analysis of such data, machine learning algorithms have shown to be useful for identifying key molecular signatures, discovering potential patient stratifications, and particularly for generating models that can accurately predict phenotypes. In this review, we first discuss how dysbiosis of the intestinal microbiota is linked to human disease development and how potential modulation strategies of the gut microbial ecosystem can be used for disease treatment. In addition, we introduce categories and workflows of different machine learning approaches, and how they can be used to perform integrative analysis of multi-omics data. Finally, we review advances of machine learning in gut microbiome applications and discuss related challenges. Based on this we conclude that machine learning is very well suited for analysis of gut microbiome and that these approaches can be useful for development of gut microbe-targeted therapies, which ultimately can help in achieving personalized and precision medicine.

## Introduction

**Fig. 1:**
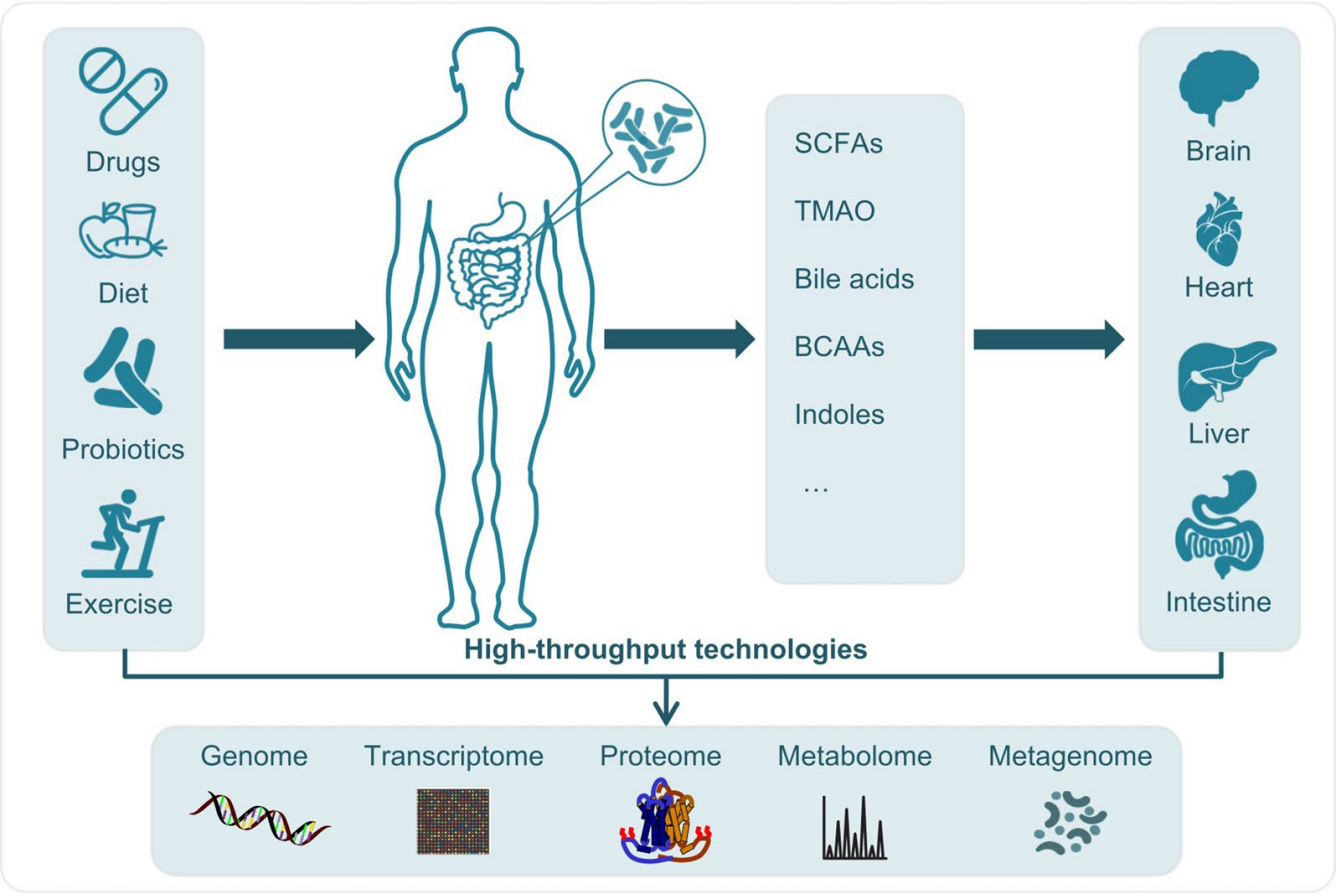
Complex interplay between the gut microbiome and human metabolism. A number of factors, including genetics, age, diet, drugs, probiotics and exercise could influence the gut microbiome. The effects of the gut microbiome on human health have been potentially mediated by the microbe-derived metabolites, such as short-chain fatty acids (SCFAs), trimethylamine N-oxide (TMAO), bile acids, indoles and branched-chain amino acids (BCAAs). The microbial metabolites could interact with different human organs, including brain, heart, liver, intestine and adipose tissue, which will hence affect human metabolism. Due to the complex interactions, high-throughput technologies have been widely applied to generate multi-omics data, including gut metagenomics, host genomics, metabolomics, proteomics, and transcriptomics from different human tissues, which provides more insights into the associations between the gut microbiota and the host

The human intestine is colonized by a vast number of commensal microorganisms referred to as the gut microbiota, composed of over 10^14^ bacterial cells whose collective genome contains 100 times more genes than the human genome [1–3]. Previous studies have demonstrated that dysbiosis of the human gut microbiota plays critical roles in various diseases, such as diabetes [4–6], obesity [7–10], inflammatory bowel disease (IBD) [11, 12], liver diseases [13, 14], neurological disorders such as autism spectrum disorder (ASD) [15, 16], cardiovascular diseases (CVD) [17–19] and colorectal cancer (CRC) [20]. To understand the associations between the gut microbiota and human diseases, next-generation sequencing technologies, including amplicon-based and whole genome shotgun sequencing, have been widely applied to characterize the microbial communities and their functional capabilities (Fig. 1). Metagenomics data from recent studies, including the Metagenomics of the Human Intestinal Tract (MetaHIT) consortium [21, 22] and the Human Microbiome Project (HMP, including two phases HMP1 and HMP2) [11, 23, 24], the TEDDY study [6, 25], have enriched our knowledge of the human gut microbiota and its impact on human physiology. Subsequently, comprehensive sequence resources of the human gut microbiome have been established, including unprecedented numbers of genomes and genes, such as the Integrated Gene Catalog (IGC), the Unified Human Gastrointestinal Genome (UHGG) and Protein (UHGP) catalogs and identification of 204,938 genomes from 4,644 gut microbes [2, 3, 26, 27]. With the development of high-throughput technologies, increasing studies have started to consider longitudinally personalized multi-omics profiling, including metabolomics, proteomics, genomics and transcriptomics from different human tissues (Fig. 1), which has enabled a more complete picture of human metabolism and provided more insights into interactions between the gut microbiota and the host [11, 23, 28, 29].

A large number of studies have indicated that factors including genetics or ethnicity [30, 31], age [32, 33], diet [34, 35], drugs [36], geography [37], and exercise [38] could influence the gut microbiome (Fig. 1), which may confound the associations between the microbiota and human diseases. Rothschild et al. demonstrated that the gut microbial composition is shaped predominantly by environmental factors rather than host genetics [39]. Due to these intricacies, it is critical to develop advanced computational methods that are efficient to extract key information from huge, heterogeneous and complex multi-omics data. Machine learning (ML) holds great promise to explore and integrate multi-omics data for discovering hidden patterns and for generating models that can accurately predict phenotypes [40–46]. Meanwhile, potential biomarkers related to human diseases could be identified through interpretable models [47–49], thus allowing us to gain novel insights into diseases and further propose potential therapeutic strategies. Especially, deep learning has achieved tremendously successful applications in various fields, such as AlphaGo [50] and AlphaFold [51]. Also, an increasing number of studies have applied deep learning approaches to analyze the gut microbiome [52–54].

In this review, we first introduce the human gut microbiome and its associations with human diseases. Moreover, we describe the main categories, general workflow and multi-omics integration strategies of ML. Finally, we summarize the recent progress of ML applications as well as discuss the challenges and future perspectives for analysis of gut microbiome data.

## The gut microbiota and human diseases

Many studies have suggested that the dysfunction of the gut microbiota contributes to the onset and progression of human diseases as reported recently [55–57]. For example, obesity is associated with a greater Firmicutes/Bacteroidetes ratio [10], and more recently Thingholm et al. suggested that individuals with obesity show different gut microbial composition including decreased *Akkermansia* and *Faecalibacterium*, compared with healthy individuals [58]. Zhong et al. also observed distinct differences in the gut microbiota of prediabetic individuals including reduced levels of *Roseburia hominis* and *Faecalibacterium prausnitzii*, and elevated levels of *Escherichia coli* [59]. Similarly, patients with IBD and Crohn’s disease have a reduced diversity or a general dysbiosis of the intestinal microbiota, including a reduced complexity of the phylum Firmicutes with decreased levels of *Faecalibacterium prausnitzii* and *Bifidobacterium adolescentis* [60–62]. Yu et al. found that several species including *Parvimonas micra* and *Solobacterium moorei* and 20 microbial gene markers are significantly associated with CRC by metagenomic analysis [20]. By analyzing multiple cross-region cohorts, Ren et al. observed alterations of the gut microbiome in patients with early liver cancer, such as increased diversity within the phylum Actinobacteria, depleted butyrate-producing genera and enriched lipopolysaccharide-producing genera [14].

### Microbe-producing metabolites linked to human diseases

By investigating the overall metabolic potential of the human gut microbiota, Visconti et al. found that microbial metabolic pathways have over 18,000 significant associations with blood and fecal metabolites [63], whereas species show less than 3,000 associations. This study indicated the importance of disentangling the microbial metabolic potential, which might unveil the underlying mechanism in relation to human diseases. To examine relationships between the blood metabolome and the gut microbiota, Wilmanski et al. predicted the alpha diversity of the gut microbiota based on a set of 40 plasma metabolites [49]. Out of the 40 metabolites, 13 are microbe-derived metabolites including imidazole propionate, secondary bile acids, trimethylamine N-oxide (TMAO) and indole propionate, which are linked to CVD risk and T2D (Fig. 1). This study implicates that the contribution of the gut microbiota to human diseases are possibly mediated by bacterial-derived metabolites. In a cross-sectional study, Kurilshikov et al. showed that plasma levels of short-chain fatty acids (SCFAs) from the gut microbial fermentation of fibers were associated with inflammation and CVD risk [64]. Moreover, Pedersen et al. identified *Prevotella copri* and *Bacteroides vulgatus* as the main drivers, which induced insulin resistance via the production of branched-chain amino acids (BCAAs) [22]. These demonstrate that the common cardiometabolic disorders could be regulated by microbial metabolites.

In addition, Chu et al. discussed the microbe-derived metabolites that potentially affect the non-alcoholic fatty liver disease (NAFLD) progression, including bile acids, SCFAs and TMAO [13], which implies the involvement of the gut-liver axis in the development of NAFLD (Fig. 1). By transplanting gut microbiota from ASD patients into germ-free mice, Sharon et al. found that the microbiota caused autistic behaviors through the production of neuroactive small molecules including 5-aminovaleric acid [15], indicating the role of the gut-brain axis in the pathophysiology of ASD (Fig. 1). By integrating metabolomic and metagenomic data, Franzosa et al. identified a number of associations between IBD-related species and metabolites including caprylic acid, which provides an insight into the possible mechanism underlying dysfunction of the gastrointestinal tract [12] (Fig. 1). As discussed in previous reviews [13, 65–67], these microbe-derived metabolites may activate or de-activate various signaling pathways and thereby contribute to human health and diseases.

### Manipulating the gut microbiota as a potential therapeutic strategy

Growing evidence has established that modifying the gut microbiota could be a potential strategy to prevent or treat diseases [68], e.g., through dietary interventions [34, 69], fecal microbiota transplantation (FMT) [70] or supplementation with probiotics and/or prebiotics [71–73] (Fig. 1). [34, 35][74]A recent study showed that the Mediterranean diet intervention alters the gut microbiome and improves health status in older people [69]. Several clinical trials have shown the efficacy of FMT in treating different diseases, such as diarrhea [75], *Clostridioides difficile* infection (CDI) [76] and hepatic steatosis [77], through altering the intestinal microbial community structure. In a randomized, double-blind, placebo-controlled trial, Sabico et al. implicated that multi-strain probiotic supplementation over 6 months significantly decreased insulin resistance and inflammation in T2D patients [78]. Also, Karamali et al. demonstrated that taking probiotic supplements in patients with gestational diabetes had beneficial effects on the glycemic control [79]. Moreover, Roberts developed an inhibitor targeting microbial enzymes [80], which significantly reduced plasma levels of microbial metabolite trimethylamine N-oxide (TMAO) associated with CVD risk [81]. Their study suggests that inhibiting the production of harmful gut microbial metabolites could offer a promising intervention target for disease treatment.

Previous studies have shown that synthetic biology could provide rational engineering of microorganisms for the prevention and treatment of diseases [82, 83]. Yuvaraj et al. genetically modified *Escherichia coli* for delivery of bone morphogenetic protein 2 that induces effective apoptosis in an in vitro model of CRC [82], which suggests that the strategy might be feasible for the treatment of CRC patients. In a recent study, the probiotic *Saccharomyces boulardii* was engineered to constitutively secrete an antibody that potently neutralized toxins related to CDI in mouse models [83]. Thus, this yeast immunotherapy has the potential as a strategy for treatment of patients with CDI. Furthermore, the increasing development of synthetic biology enables the construction of synthetic microbial consortia as reviewed previously [84], which extends the rational engineering from single microorganism to a multicellular microbial community. Therefore, the development of gut microbe-targeted therapies by reversing dysbiosis of the microbiota, inhibiting microbial enzymes or genetically engineered probiotics, has been suggested to be feasible and efficacious. Particularly, ML has been widely used to identify microbial biomarkers for evaluation of disease risk or for designing gut microbe-targeted therapies.

## Machine learning

**Fig. 2:**
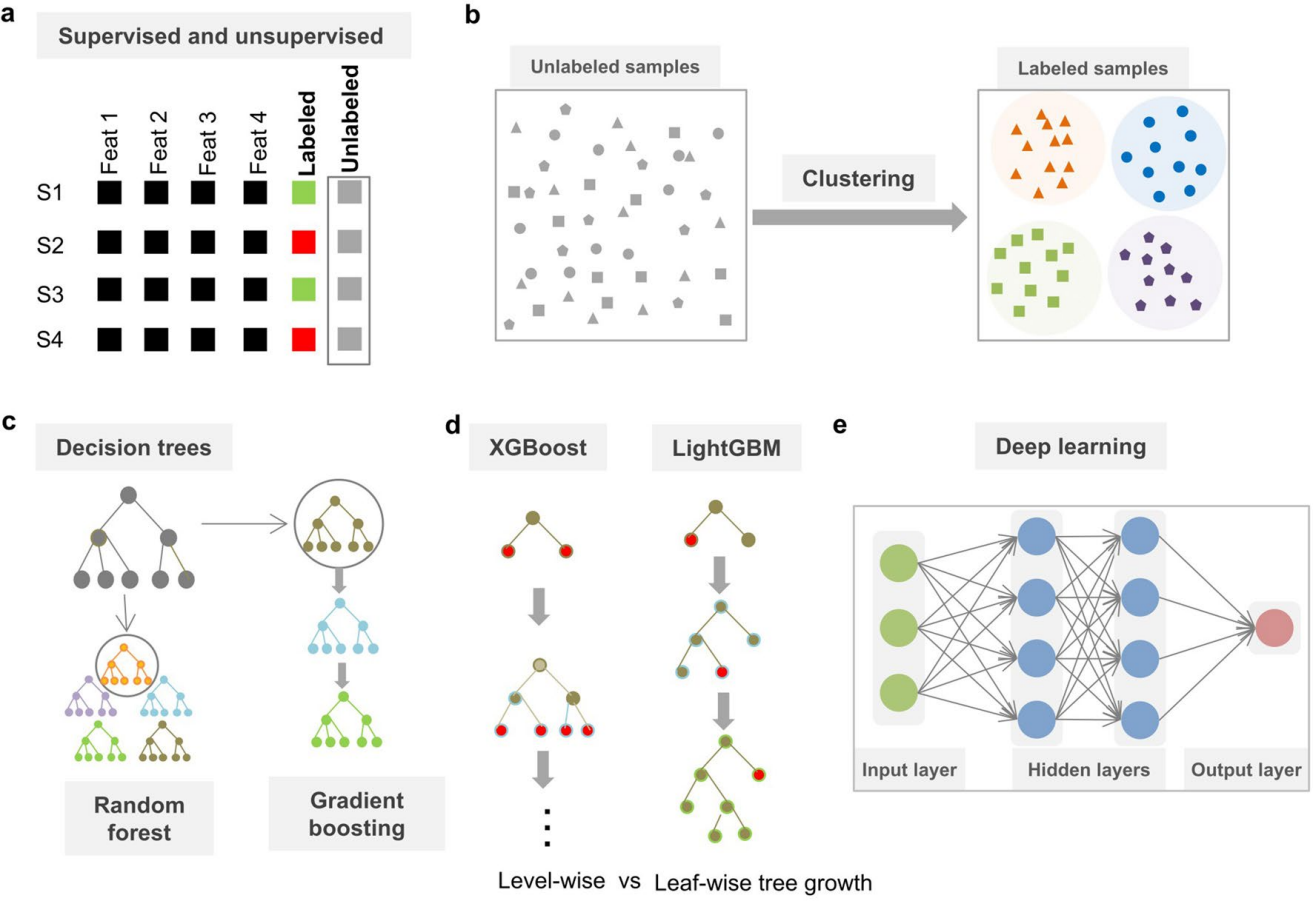
The main categories of machine learning algorithms for analysis of the gut microbiome. **a** Characteristics of supervised and unsupervised learning. Supervised learning can learn a function to map the independent variables (features) with the known dependent variable (called label) from a training dataset, whereas unsupervised learning methods purely learn and discover novel hidden patterns from the given dataset without the dependent variable, i.e., unlabeled as in the box. S1-S4 in row and Feat1-Feat4 in column represent different samples and features, respectively. For supervised learning, labels in various colors indicate different continuous values or classes; **b** clustering analysis. As an unsupervised learning method, it purely discovers novel patterns from a dataset based on similarities or dissimilarities between training samples. For example, here samples can be stratified into the four clusters by k-means clustering that minimizes the within-cluster sum of squares. Each color denotes one cluster; **c** relationships between decision trees, RF and gradient boosting; **d** comparison of XGBoost and LightGBM. XGBoost splits the tree level-wise (also called depth-wise), while LightGBM splits the tree leaf-wise. The decision node in red color represents the node can be split into children node at each layer; **e** deep learning. In a deep neural network architecture, multiple (here two) hidden layers (blue color) are connected in a cascade fashion between input (green color) and output layers (red color). Each of these layers takes input from its previous layer and transforms the data into a more abstract form as an output for next layer

ML is a branch of artificial intelligence that automatically learns and improves from input data without being explicitly programmed. ML algorithms are mainly classified into unsupervised learning and supervised learning, which have been widely applied for analysis of gut microbiome. Unsupervised learning methods purely learn and discover novel hidden patterns from given datasets without known dependent variables, and are therefore referred to as data-driven prediction (Fig. 2a). Two main categories of unsupervised learning algorithms are dimension reduction and clustering analysis. The prominent dimension reduction methods include principal components analysis (PCA) [85], principal coordinate analysis (PCoA) [86] and t-distributed stochastic neighbor embedding (t-SNE) [87], which have been widely used for omics data visualization by extracting a set of principal variables from high-dimensional feature space [88, 89]. Clustering algorithms, including k‑means clustering [90], hierarchical clustering [91] and self-organizing map (SOM) [92], are frequently implemented to partition or stratify a set of objects into multiple groups (clusters) based on similarities or differences (Fig. 2b). Particularly, clustering analysis has also been applied to the identification of novel patterns in gut microbiota studies [93], such as discovering enterotypes of the human microbiota [17, 94] and co-abundance gene groups [95].

In contrast to unsupervised learning, supervised learning approaches learn and infer a function from input data, which is typically comprised of independent variables (i.e., features) and dependent variables across all samples (Fig. 2a). For supervised learning, the known dependent variables in a training dataset are used to develop an ML model, which is potentially capable to predict the outcomes of new samples. When the dependent variables are categorical, the ML model can be applied for classification tasks [5, 96]. As the dependent variables are continuous, they can be used for regression tasks [49, 97]. Several early reports have discussed and compared commonly used supervised ML algorithms [98], such as support vector machine (SVM) [99], Naïve Bayes (NB) [100], and K-nearest neighbor (KNN) [101]. Particularly, decision trees-based ensemble learning methods have been widely applied in gut microbiota studies, such as random forest (RF) [5, 96, 102], light gradient boosting machine (LightGBM) [48, 103] and extreme gradient boosting decision trees (XGBoost) [47, 104, 105], due to their powerful performance, ease of use and interpretability. In the following, we introduce the decision tree-based ML algorithms in detail, including RF [102] and gradient boosting [106], in comparison to deep learning [107], which has been recently utilized in the gut microbiome [52–54]. Also, the pros and cons of these algorithms are explained and compared.

### Decision tree

Decision tree algorithm is a common used predictive modeling approach [108], which uses a tree model to infer the target variable from input features and provides easy interpretations of the trained model. In the structure of a classification tree (Fig. 2c), leaves represent class labels and branches represent conjunctions of features that lead to those class labels. The decision tree builds the basis for other ensemble learning methods, such as RF and gradient boosting.

### Random forest

RF is a type of ensemble ML algorithm [102], also called bootstrap aggregation or bagging, which has been widely applied in gut microbiota studies [5, 96, 102]. In RF, multiple decision trees models are trained simultaneously on a random subset of the training data and then use an aggregation of their predictions to obtain the final prediction outcome (Fig. 2c). By combining many weak learners, RF is able to improve the performance of a single decision tree and make it more invulnerable to overfitting.

### Gradient boosting

In contrast to RF where all decision trees are constructed independently of each other at the same time, gradient boosting is another type of ensemble ML algorithm [106], where each weak learner is sequentially trained and improved based on the previous one until a good fit to the training data is obtained (Fig. 2c). Typically, the decision tree is chosen as base learner and the gradient descent algorithm is used to minimize the loss function during the training phase. Then, the gradient boosting generates a final prediction based on a weighted combination of the multiple decision trees’ predictions. XGBoost [104] and LightGBM [103] have recently been developed to perform the gradient boosting framework based on the decision tree. The main difference between the XGBoost and LightGBM algorithms is how the tree grows. In other words, XGBoost splits the tree level-wise (also called depth-wise) like other boosting algorithms, while LightGBM splits the tree leaf-wise (Fig. 2d). The result is that LightGBM could cut down more loss than the level-wise algorithm XGBoost when splitting the same leaf. In addition to better accuracy, the training speed of LightGBM model is faster with higher efficiency.

Both RF and gradient boosting models can be trained on different types of data structures, even a combination of categorical and numerical variables or multi-omics data that have been accumulated in gut microbiota studies. In addition, decision tree-based algorithms are not completely black box systems, since they can identify critical features by evaluating and scoring their importance, which could shed light on which factors are associated with the predicted task. Therefore, these tree-based methods are very suitable to tackle different problems in biological research.

### Deep neural network

If a training dataset includes different types of data, such as clinical data, lifestyles, metagenomics, metaproteomics and metatranscriptomics, it is challenging to deal with the high-dimensional and heterogeneous features using the traditional ML methods, which are dependent on the well-defined, engineered and hand-tuned features as inputs to make reasonable predictions. However, deep learning has proved to successfully handle and integrate multi-omics data with high dimensionality and relatively few samples [109–112]. As a subfamily of ML methods, deep learning is a deep neural network (DNN) with many hidden layers [107]. In a DNN architecture as illustrated in Fig. 2e, two or more hidden layers are connected in a cascade fashion between an input layer and an output layer. The data is transferred directly from the input layer to the first hidden layer. Each of the hidden layers takes input from its previous layer and transforms the data into a more abstract form, which is finally processed in the output layer leading to the predictive outcome. Using the DNN, raw features can be automatically extracted and learned for a desired outcome. Moreover, DNN is highly flexible and can easily adapt to new tasks. However, this type of network architecture usually generates many hyperparameters, which requires larger amounts of data to learn from training, compared to traditional ML methods.

In addition to the above unsupervised and supervised learning algorisms, semi-supervised and reinforcement learning have been successfully applied in biological studies [113–116]. Compared to supervised learning based on the fully labeled data, the semi-supervised learning algorithm uses partly labeled data. The algorithm first applies unsupervised learning to label the unlabeled data and then uses supervised learning to train predictive models. Therefore, semi-supervised learning methods are very useful when parts of datasets are not labeled. Unlike supervised learning, reinforcement learning algorithms do not use labeled data, but instead use a number of rules, which guide actions to solve a predefined problem in an iteratively self-teaching way without any input of data. The probably best-known application of reinforcement learning is AlphaGo masters the classic game of Go [50].

### The general workflow of machine learning modeling

**Fig. 3:**
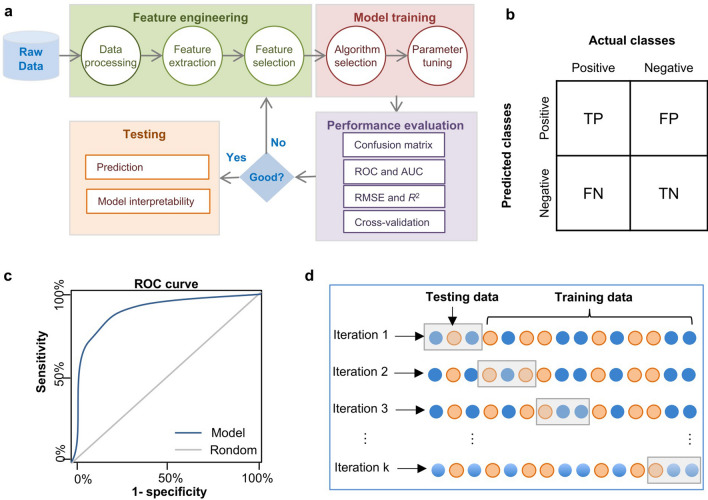
The general workflow of machine learning modelling. **a**, The entire pipeline of modeling commonly consists of four steps, including feature engineering, model training and optimization, performance evaluation, model application and explanation; **b**, Confusion matrix. It summarizes and visualizes four possibly predictions from a binary classification model, including true positive (TP), false positive (FP), true negative (TN), false negative (FN); **c**, ROC curve. It plots sensitivity against ‘1- specificity’ at varied classification thresholds of the predictive model; **d**, k-fold cross-validation. The original samples are randomly split into k subsets with equal size. When one round of cross-validation is implemented, a predictive model is trained using the k-1 subsets and validated using the single remaining subset

Although numerous supervised ML algorithms have been developed, the whole pipeline of modeling commonly consists of four steps: 1, feature engineering; 2, model training and optimization; 3, performance evaluation; 4, testing of the optimal model (Fig. 3a). The excellent performance of an ML model lies to a great extent on the quality of data used for training the model. Thus, it is essential to perform feature engineering first, which is involved in data pre-processing, feature extraction and feature selection processes (Fig. 3a). Data pre-processing includes proper cleaning, normalization and transformation. Feature extraction is intended to build a feature vector representing a decreased number of variables from raw measured data, which is required to contain sufficient relevant information from the raw data. This can facilitate subsequent training steps. But lots of features in the dataset might be still uninformative and irrelevant for constructing a predictive model. For example, model construction with extremely large amounts of variables (genes, proteins, metabolites, etc.) requires extensive computing power and memory, and easily leads to overfitting. Thus, feature selection is important to obtain an optimal and non-redundant subset of the initial features [117], which is critical for fast model training, improved performance and even better model interpretation.

Moreover, confusion matrix, receiver operating characteristic (ROC) and assessment metrics including area under ROC curve (AUC), root mean square error (RMSE), coefficient of determination (*R*^2^), are frequently applied to evaluate model performance for classification or regression tasks (Fig. 3a). A confusion matrix is an n-dimensional table, where n is the number of predicted classes in a classification task. Given a binary classification (n = 2), the predictive outcomes are either positive or negative, and the confusion matrix briefly summarizes and visualizes four possibly predictions from a classification model (Fig. 3b), including true positive (TP; both the predictive and actual values are positive), false positive (FP; the predictive outcome is positive, but the actual value is negative), true negative (TN; both the predictive and actual values are negative), false negative (FN; the predictive outcome is negative while the actual value is positive). Several important assessment metrics based on the confusion matrix are introduced as follows: (1) Accuracy = (TP + TN)/(TP + TN + FP + FN), representing the percentage of classes that are predicted correctly; (2) Precision = TP/(TP + FP), referred to positive predictive value; (3) Sensitivity = TP/(TP + FN), called the true positive rate (TPR) or recall, representing the percentage of actual positive cases being predicted correctly; (4) Specificity = TN/(TN + FP), called as true negative rate (TNR), representing the percentage of actual negative cases being predicted correctly. A ROC curve plots sensitivity (TPR) against ‘1-specificity’ (false positive rate, FPR) at varied classification thresholds of the predictive model (Fig. 3c). If the ROC curve of a classifier is closer to the top-left corner, it suggests a better performance. In comparison, the ROC curve of a random classifier ususally lies along the diagonal, indicating poor performance, i.e., the classifier is similar to random choice. The area under ROC curve (AUC) summarizes the model performance into a single measure ranging from zero to one, which is widely used to compare the performance of different classifiers.

In addition, k-fold cross-validation has been widely used to evaluate model performance, where the original samples are randomly split into k subsets with equal size at first (Fig. 3d). When one round of cross-validation is implemented, a predictive model is trained using k-1 subsets (i.e., training set) and validated using the single remaining subset (i.e., test dataset). This step is iterated k times, where each of the k subsets is used as a test dataset. Then, the k validation outcomes are summarized into a single metric (e.g., averaged) for assessing whether the predictive model is accurate and robust. For most ML methods, the training process includes iterations of model parameters tuning and feature engineering until the model performance cannot be improved further (Fig. 3a). The performance of multiple different approaches can be benchmarked and then the one or two best models can be selected. Finally, the model can be applied to make predictions on new data. Notably, disease-related biomarkers can be simultaneously identified by model interpretability in previous microbiota studies [47, 48] (Fig. 3a), which allows us to gain biological insights into the data. Overall, the above processes can impact the model performance and thus should be taken into account when implementing a ML algorithm in gut microbiome research.

### Integrative strategies for analysis of multi-omics data using machine learning

High-throughput technologies have been widely applied to profile the microbial ecosystem and human metabolism, which has led to an explosion of multi-omics data, such as metabolomics, transcriptomics, metagenomics, etc. (Fig. 1) Single omics data analysis normally provides a partial view on the complexity of biological system, and integrative analysis of multi-omics data is therefore extremely critical to disentangle associations between the gut microbiota and human diseases. Accumulated evidence has shown that ML holds great promise to analyze and integrate heterogeneous data in gut microbiota studies [53, 118–120].

**Fig. 4:**
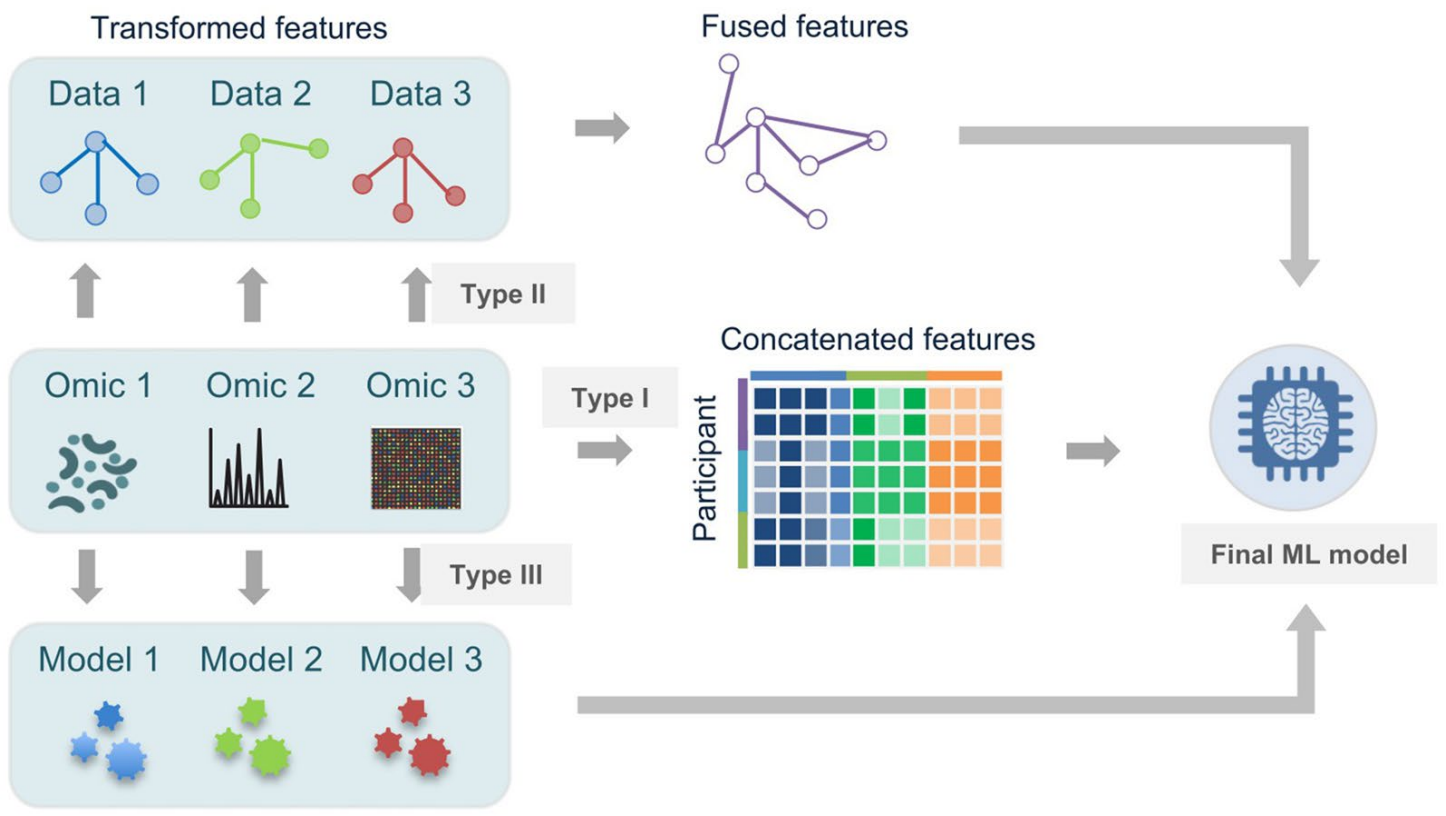
Integrative strategies of multi-omics data in machine learning. Machine learning methods for integration of multi-omics data are mainly categorized into three types. The first type of integrative strategy combines directly each omics data into one large matrix before training machine learning model. The second type of integrative strategy firstly transforms each omics data into an intermediate form that then are combined for further training. The third type of integrative strategy firstly trains machine learning model separately using each omics data and then combines the predictive outcomes of the trained individual models to generate a finalized model

Integrative methods of multi-omics data in machine learning can mainly be categorized into three types [42, 46]. The first type of integration strategy combines directly each omics data into one large matrix before training machine learning model, where the data integration happens at early stage (Fig. 4). The trained model is also referred to as a data-driven model [118, 119], which has been widely applied in gut microbiota studies [12, 118, 119]. For instance, Zeevi et al. integrated multi-dimensional data including blood parameters, dietary habits, anthropometrics, physical activity and gut microbiota by a gradient boosting regression model, which predicted accurately postprandial glucose responses to real-life meals [118]. Moreover, to integrate and analyze metabolomic and metagenomic data, Franzosa et al. trained RF models based on the microbial species profiles, metabolites abundances and their combination to classify IBD patients and subtypes [12]. The predictive models were then validated on an independent cohort with AUC ranging from 0.86 to 0.89. Li et al. also constructed RF classifiers based on gut microbiota and metabolite abundances to discriminate pre-hypertensive and hypertensive patients from healthy controls with AUC of ~ 0.9 [119]. Their results reveal that gut microbiota dysbiosis contributes to the development of hypertension. Additionally, Gao et al. demonstrated that integration of microbial pathways with serum metabolites as a predictor of 30-day mortality in patients with alcoholic hepatitis performed better than using single omics data as predictors [121].

The second type of integrative strategy firstly transform each omics data into an intermediate form, such as a graph or a kernel matrix[122–124], a learned latent representation of DNN [109–112] and a set of hidden factors [125], which then can be combined together for further training and analysis (Fig. 4). This type of data integration happens at intermediate stage before or during training of the model. For example, recently Hira et al. used DNN-based variational autoencoder (VAE) to extract learned latent features from high dimensional data, and then performed an integrated multi-omics analysis of ovarian cancer using the compressed features [111]. Moreover, Tsubaki et al. firstly learned an individual representation for compounds and proteins, using a graph neural network for compounds and a convolutional neural network for proteins [109]. Then the inferred representations were integrated and used to predict compound–protein interactions. Additionally, Argelaguet et al. proposed an unsupervised computational framework for inferring latent factors that represent the principal sources of variation in multi-omics data sets [125]. The learnt factors were further used to classify sample subgroups.

The third type of integrative strategy firstly trains machine learning model separately using each omics data, which then combines the predictive outcomes of the trained individual models to generate a finalized model (Fig. 4), e.g., ensemble learning methods [126] RF and gradient boosting[127]. This type of data integration happens at late stage after training the initial model using each omics, which is very proper for application of ML in multi-site trials with heterogeneous datasets. All these three integrative methods have been widely applied to analyses of biological data as discussed in the previous review [128].

## Application of machine learning for analysis of gut microbiome data

ML methods have been applied successfully for analysis of gut microbiome data, including discovery of hidden patterns and accurate predictions of phenotypes. Table 1 summarizes the main applications of ML approaches in the field of gut microbiome.

**Table 1:** Summary of machine learning applications in gut microbiome studies.

Category	Predictive task	Algorithm	Performance	Sample size	Data type	Data source	Reference
Phenotypic prediction	T2D risk	SVM; mRMR	AUC = 0.81	345	MG	SRA045646	[4]
		RF	AUC = 0.83	96	MG	ERP002469	[5]
		LightGBM	AUC = 0.73	1832	16 S rRNA	CNP0000829	[48]
	CRC risk	Lasso	AUC > 0.8	141	MG	ERP005534	[129]
		mRMR	AUC = 0.77	96	MG	ERP008729	[20]
	CVD risk	RF	AUC = 0.7	951	16 S rRNA	American Gut Project [130]	[19]
	IBD risk	RF	AUC > 0.86	155	MG; metabolomics	PRJNA400072; PR000677	[12]
		MetaNN	AUC = 0.89	425	16 S rRNA	PRJNA237362	[131]
	Cholera	SVM	AUC = 0.8	76	16 S rRNA	PRJEB17860	[132]
	Obesity	RF	AUC = 0.66	253	MG	ERP003612	[8, 133]
		MVIB	AUC = 0.66				[134]
	Hypertension	RF	AUC = ~ 0.9	196	MG; metabolomics	PRJEB13870	[119]
	Liver cirrhosis	DeepMicro + SVM	AUC = 0.9	237	MG	ERP005860	[54, 135]
		EPCNN	AUC = 0.95				[136]
	Alcoholic hepatitis	Logistic regression	AUC = 0.89	43	MG; metabolomics	ERP106878	[121]
Recommended therapeutics	Infliximab treatment	RF	AUC > 0.86	16	16 S rRNA	PRJEB22028	[96]
	Immunotherapy	RF	AUC = 0.6	103	MG	PRJEB22893; PRJNA399742	[137]
Personalized nutrition	Glucose response	Gradient boosting	PCC = ~ 0.7	800	16 S rRNA	PRJEB11532	[118]
Stratification	Enterotypes	PAM Clustering	3 clusters	154	16 S rRNA	NCBI SRA	[94]
			2 clusters	25	MG	–	[17]
			2 clusters	98	16 S rRNA	SRX020773	[74]
	Identification of CAGs	Canopy-based clustering	7,381 CAGs	396	MG	ERP002061	[95]

These applications have been mainly classified into phenotypic prediction, precision medicine and stratification of population

SRA*, SRX*, ERP* and PRJ* from NCBI Short Read Archive (SRA) or EMBL European Nucleotide Archive (ENA); CNP* from Sequence Archive of China National GeneBank (CNGB); Metabolomics data PR000677 from the National Institutes of Health Common Fund’s Metabolomics Data Repository and Coordinating Center

*PCC* Pearson correlation coefficient of predicted and measured values,* PAM* partitioning around mediods,* CAGs* Co-abundance gene groups,* MG* metagenomics

### Phenotypic prediction and biomarker discovery

**Fig. 5:**
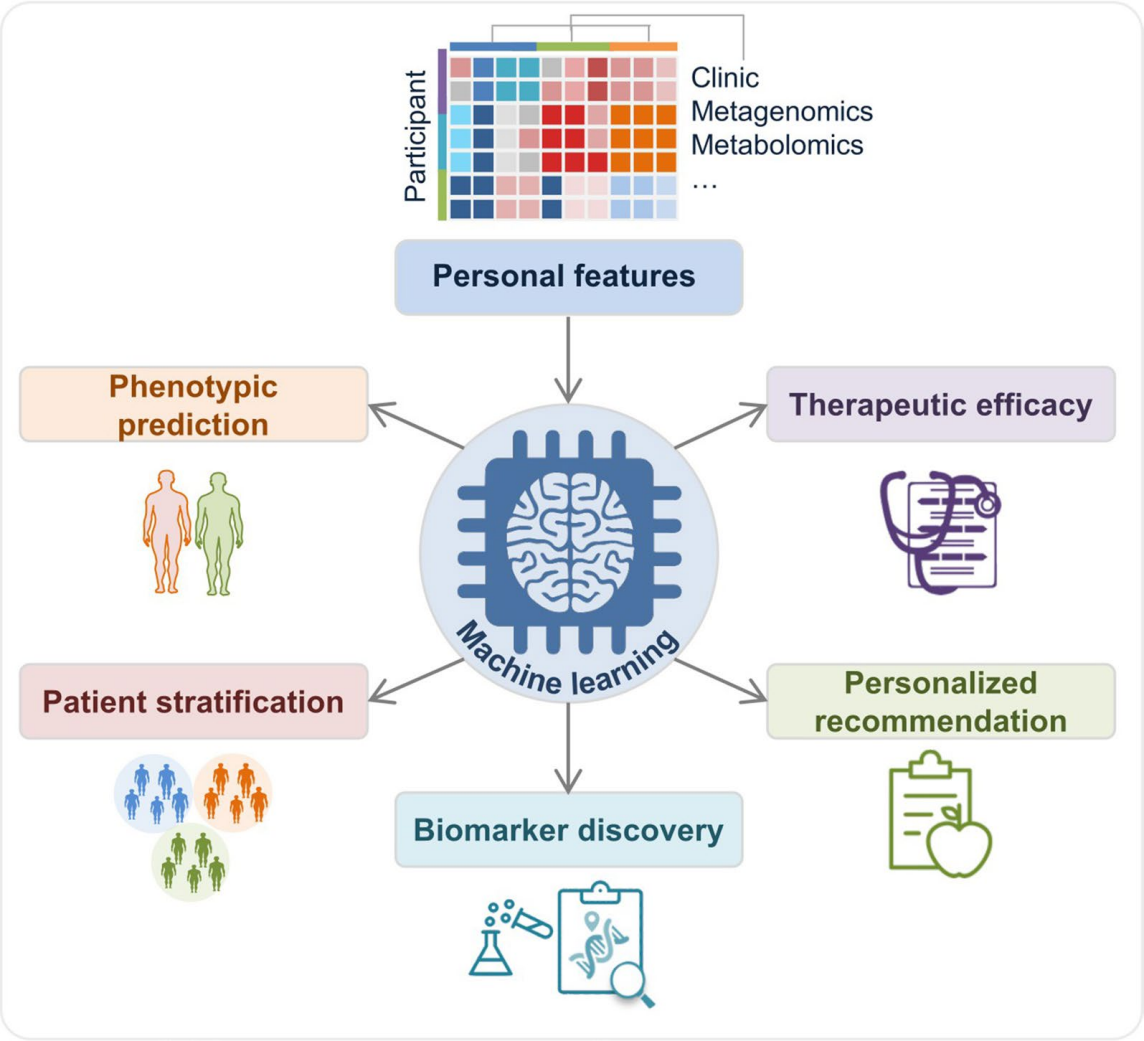
Machine learning applications in the gut microbiome. Personalized features, including clinic parameters, gut microbial signatures and features from multi-omics of human host, are used to train models for different applications, such as phenotypic prediction, patient stratification, biomarker discovery, treatment outcome evaluation, personalized treatment and nutrition

Emerging evidence has substantiated the potential ability of the gut microbiome for predicting disease states (Fig. 5), such as CRC [20, 129], CVD [19], T2D [4, 5, 48], IBD [12] and cholera [132]. With the lasso logistic regression classifiers, an early study identified key taxon including *Fusobacterium* species and *Peptostreptococcus stom*atis, which distinguished CRC patients from control populations [129]. Moreover, Yu et al. [20] used the minimum redundancy–maximum relevance (mRMR) feature selection method [138] and identified an optimal set of 20 microbial genes that were predictive of CRC status. Out of them, four discovered genes were then validated and could distinguish CRC patients from controls in different ethnical cohorts. Their results highlight the potential for applying ML as an effective tool to identify microbial biomarkers for early diagnosis of CRC (Fig. 5). In addition, Aryal et al. used five ML algorithms to predict CVD risk based on gut microbiota composition [19]. The trained predictors achieved a testing AUC of ~ 0.7, showing the potential of microbiome-based ML model for a non-invasive diagnosis of CVD.

To explore the potential classification ability for T2D patients by the gut microbiota, Qin et al. developed a T2D classifier system using gut microbial gene markers and mRMR feature selection method [4, 138]. The predictive results for classifying T2D individuals showed high accuracy with an area under the ROC curve (AUC) of 0.81. Moreover, Karlsson et al. developed RF models based on the microbiota composition of a Swedish cohort to classify T2D status with AUC ranging from 0.71 to 0.85 [5]. Recently, Gao et al. used an interpretable ML framework and identified robust T2D-related gut microbiome features in cross-sectional analyses of three Chinese cohorts [48]. By constructing a microbiome risk score (MRS) with 14 identified features, they found that the MRS was associated with future glucose increment and several gut microbiota-derived metabolites. In addition, using baseline gut microbiota data, Midani et al. constructed SVM models to predict *Vibrio cholerae* infection [132] and they found that the susceptibility to cholera was correlated with decreased abundances of the phylum Bacteroidetes.

The high dimensionality and relatively low sample sizes of typical gut microbiome data can lead to the curse of dimensionality that challenges traditional ML methods [139]. To transform high-dimensional microbiome profiles into a low-dimensional representation, a recent study developed a deep representation learning framework (DeepMicro) [54], which uses different autoencoders to compress the microbial features. Then the learned representations were utilized for various disease predictions using classification algorithms including SVM and RF. Their results show that the DNN-based framework accelerates the model training process and improves the model performance of disease prediction. Moreover, several DNN-based architectures integrating with the phylogenetic tree, such as EPCNN [136], Ph-CNN [140], PopPhy-CNN [141], have been developed, which show an improved performance.

### Precision medicine for recommended therapeutics and nutrition

Biological complexity usually results in high interpersonal variability in the response to identical medical treatment or diet intake [142], which suggests that universal intervention may have limited efficacy for a specific disease. For instance, previous studies have shown that individuals have significant differences in postprandial blood glucose responses to identical meals. To take potential factors that may influence the personal response into consideration, Zeevi et al. devised a gradient boosting regression model to predict accurately postprandial glucose responses from dietary information, gut microbiome, etc. [118]. Based on this model, predicted dietary intervention can significantly lower postprandial responses. Their results suggest that ML could be critical in the development of personalized nutrition combined with the gut microbiome (Fig. 5), which would be efficient for controlling all kinds of diseases. In addition, to evaluate response to infliximab treatment in patients with IBD, Zhou et al. built RF models based on gut microbiota profiles, which showed accurate prediction of the treatment effectiveness [96]. They found a significant increased abundance of *Clostridiales* in patients responding to the infliximab treatment. Also, a meta-analysis study predicted response to cancer immunotherapy in patients with metastatic melanoma by a RF classifier based on gut microbial features [137]. These indicate that the gut microbiota could offer predictive biomarkers for evaluation of treatment efficacy (Fig. 5).

### Patient stratification and classification of disease subtypes

Due to intricate biological regulation, a number of prevalent diseases have shown large heterogeneity between individuals, such as T2D [143] and cancer [144]. Thus, it’s critical to correctly stratify heterogeneous patients with a specific disease into several subgroups based on clinical and multi-omics data (Fig. 5), as this may discover true disease subtypes and hereby enable personalized medicine. As a good example, Ahlqvist *et a*l. performed k-means clustering to categorize adult-onset diabetes into five subgroups with similar characteristics [143], which can be discriminated by clinical biomarkers for each subgroup. Consequently, five different T2D subtypes were proposed, which could guide differential T2D treatments. In addition, a recent study identified six distinct clusters of prediabetes using clustering analysis and clinical data [145]. These results suggest that pathophysiological heterogeneity manifests prior to diagnosis of T2D, which could guide preventive and therapeutic strategies for T2D. What’s more, a previous study identified novel subtypes of CRC using a deep learning-based method and multi-omics data sets [144]. Their method could learn latent factors that depict data in lower dimensions and explain the variability of molecular profiles.

Interestingly, numerous studies indicate that the human gut microbiota can be stratified into three robust clusters (referred to as enterotypes) that are distinguished primarily by genus levels of *Bacteroides*, *Ruminococcus* and *Prevotella* [17, 94]. Wu *et a*l. reported that enterotypes are significantly associated with long-term diets, particularly protein and animal fat (*Bacteroides*) and carbohydrates (*Prevotella*) [74]. There is therefore strong evidence for that gut microbiome-based stratification could guide personalized interventions to benefit human health [146].

### Challenges and future perspectives for machine learning in the gut microbiome

ML is a promising tool to analyze the gut microbiota related to multi-omics data and identify microbial biomarkers for noninvasive evaluation of disease risk or for designing gut microbe-targeted therapies. Using ML, we can also stratify patients based on the gut microbiota and then apply personalized therapeutics or nutrition. [2, 26, 27, 147] However, current applications of ML for analysis of gut microbiome data still fall behind other scientific areas to some extent. There are several challenges to overcome in the field of gut microbiome.

[5, 48, 54, 94]High-dimensional and heterogeneous data with extremely large amounts of molecular features (genes, species, metabolites, etc.) but relatively small sample size makes it difficult to develop robust and accurate prediction models, and easily leads to overfitting problem. To prevent overfitting, a few techniques could be useful such as using cross-validation and feature selection [117], reducing the model complexity [148], training with more data. In addition, various autoencoder-based deep learning methods have been devised to transform high-dimensional features into low-dimensional latent representations [54], which could be used for further analysis and prediction. Also, data augmentation techniques that create newly synthetic data based on existing data can mitigate the effects of over-fitting [131]. Deep learning includes lots of hyperparameters and requires large amounts of data for training. Indeed, high-throughput technologies to generate omics data are improving tremendously and the costs per sample are declining rapidly, so ML models in gut microbiota studies can be trained with expanding datasets, and might become more powerful and applicable in the future. Moreover, it is challenging to integrate multi-omics data and elucidate biological interactions between different molecular profiles that contribute to disease, although a number of ML approaches have been developed for integrative analysis of multi-omics data [53, 110, 111, 120, 125]. Particularly, all kinds of confounding factors, such as drugs, age, diet, could affect associations between the gut microbiota and a unique disease [32–36], which further make it challenging to build ML models with high accuracy and extract disease-specific signatures. Therefore, these intricate confounders should also be integrated into ML models, which could improve the model performance. Furthermore, the development of gut microbial predictive model and diagnostic biomarkers would be possibly specific to the population or region studied, and difficult to be generalized across multiple ethnicities or geographies [37].

In addition, one limitation of ML application is its standardization in conducting multi-site trials that usually generate datasets with different samples and inconsistent variables. To overcome this, the integrated ML model with the third type of strategy, e.g., ensemble learning algorithm could be a good choice. What’s more, imbalanced dataset in practice has great impacts on accuracy of the trained classifiers. To balance the classes, either more data belonging to the smaller class is required or data from the larger class is discarded. Here data augmentation technique can be used to create new data for the smaller class. Moreover, although some interoperable ML algorisms have been developed, it is still difficulty to clarify the biological mechanism underly pathogenesis of diseases.

With the accumulation of large amounts of gut metagenomic data, ML can be used to identify a large number of novel microbial genomes and proteins from uncultured species [2], forming the basis for mechanistic understanding of the gut microbiome. Based on these unexplored protein sequences, ML can be further used to predict protein structure for enzyme design or drug development [51]. Especially, ML can be applied to tailor healthy food for every person [118] and recommend therapeutics for certain patients [96], based on their gut microbiome and diet information. Moreover, ML can be applied to assist in design of probiotics and even synthetic microbial multispecies consortia. Therefore, these ML applications can ultimately help to achieve the microbiome-based personalized nutrition and precision medicine. Although all kinds of challenges facing us, the success of artificial intelligence accompanied by big data has paved the road to future applications of the gut microbiota, which could be a great opportunity to develop gut microbiota-targeted strategies for treatment and prevention of human diseases.

## Data Availability

Not applicable.
